# Supplementary material for: Health Status of Bycaught Common Eiders (Somateria mollissima) from the Western Baltic Sea
Source: Animals (Basel). 2022 Aug 8;12(15):2002. doi: 10.3390/ani12152002 (PMC9367620; doi:10.3390/ani12152002)
Supplement: Supplementary file 1 [file animals-12-02002-s001.zip › animals-1827248-supplementary.pdf]

Supplementary Materials

Health Status of Bycaught Common Eiders (Somateria mollissima) from the Western Baltic Sea

Luca A. Schick [1], Peter Wohlsein [2], Silke Rautenschlein [3], Arne Jung [3], Joy Ometere Boyi [1], Gildas Glemarec [4], Anne-Mette Kroner [4], Stefanie A. Barth [5] and Ursula Siebert [1,*]

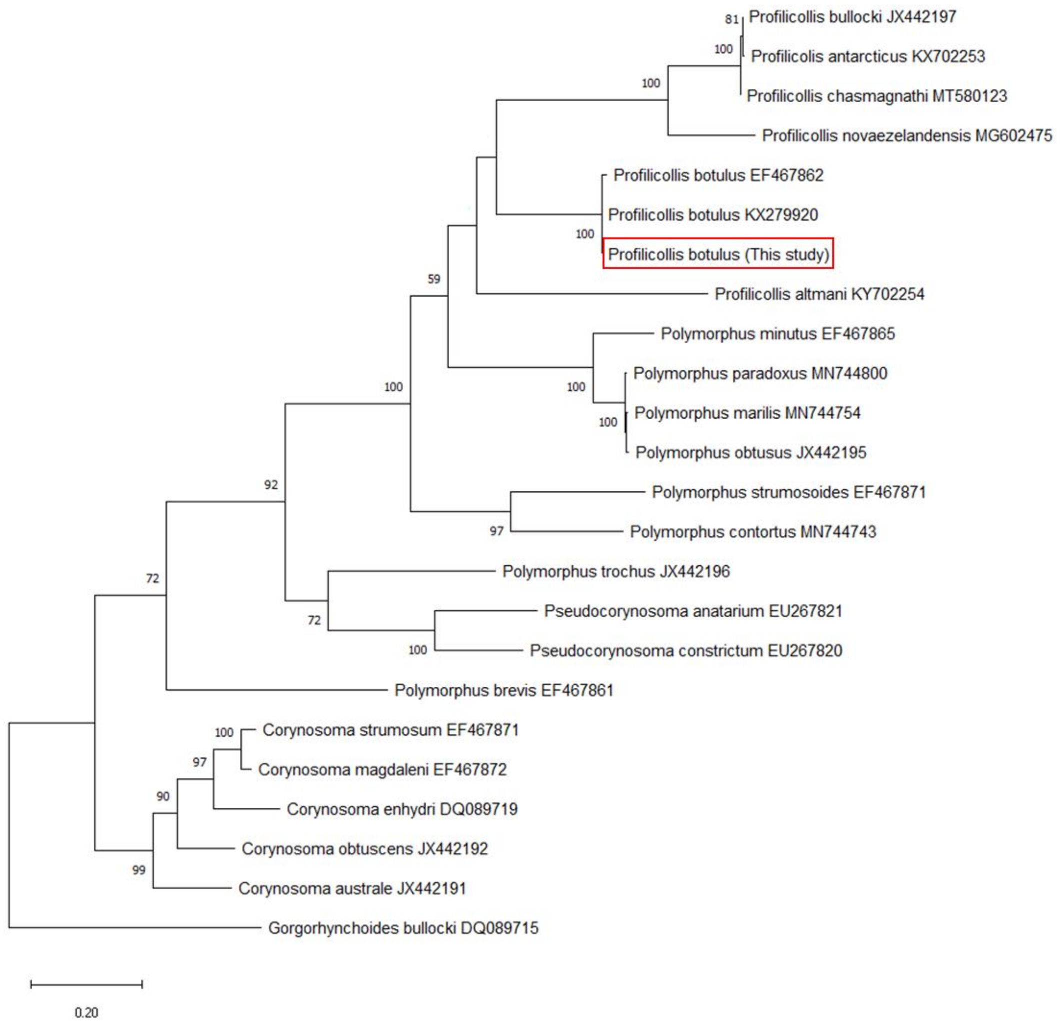

Figure S1. Maximum likelihood analysis of phylogenetic relationships of *Profilicollis botulus* in relation to other members of the Polymorphidae family using Cytochrome oxidase subunit I (COI) sequences. *Gorgorhynchoides bullocki* was designated as outgroup. Nodal support is indicated by bootstrap values (n = 1000 bootstrap replicates), values below 50% are not shown. The sequence in the red box is from this study.
